# A Rare Cause of Scalp Swelling in Infants: Two Case Reports

**DOI:** 10.7759/cureus.75632

**Published:** 2024-12-13

**Authors:** Isabel M Ribeiro, Dora Sousa, Ruben Rocha, Carmen Carvalho, Cristina Godinho

**Affiliations:** 1 Pediatrics, Centro Hospitalar Universitário de Santo António, Porto, PRT; 2 Pediatric Neurology, Centro Hospitalar Universitário de São João, Porto, PRT; 3 Neonatology, Centro Hospitalar Universitário de Santo António, Porto, PRT

**Keywords:** delayed subaponeurotic fluid collection, infant, neonates, scalp swelling, subgaleal

## Abstract

Delayed subaponeurotic fluid collection (DSFC) is an uncommon condition that causes scalp swelling in infants, usually appearing within the first weeks to months of life. Although the precise etiology is unclear, DSFC is frequently associated with instrumental or traumatic deliveries.

We report two cases of DSFC: a 12-week-old boy and a six-week-old girl, both presenting with progressive, fluctuant scalp swelling without any history of trauma. Notably, both infants had a history of vacuum-assisted delivery. Conservative management was adopted in both cases, with complete resolution of the fluid collections within four weeks.

DSFC is a benign and self-limiting condition, and early recognition is essential to prevent unnecessary investigations or interventions. This report adds to the limited literature on DSFC and highlights the importance of awareness among pediatric healthcare providers.

## Introduction

Scalp swelling in neonates is a common clinical finding with a broad differential diagnosis. When evaluating scalp swelling in infants, it is essential to consider subaponeurotic or subgaleal hemorrhage, characterized by a fluctuating mass that crosses suture lines and may enlarge rapidly, often a medical emergency. Additionally, cephalhematoma, which does not cross suture lines and is usually linked to instrumental delivery, typically resolves within weeks. Caput succedaneum, a diffuse, superficial edema that crosses suture lines, emerges shortly after birth and resolves within days [1-3].

Delayed subaponeurotic fluid collection (DSFC) is an additional cause of scalp swelling in newborns and infants, characterized by the accumulation of fluid between the galea aponeurosis and the periosteum. This is a relatively newly identified condition, with fewer than 67 cases documented in the literature [2].

While the exact etiology of DSFC remains unclear, proposed mechanisms suggest associations with birth trauma, instrumental deliveries, or the use of scalp electrodes, which may disrupt venous or lymphatic drainage or cause cerebrospinal fluid (CSF) leakage [1-3].

DSFC typically presents several weeks to months after birth in otherwise healthy infants with no recent history of trauma. Clinically, it manifests as a poorly defined, soft, fluctuant, mobile and non-tender swelling that crosses cranial suture lines, typically found over the superior occiput. Diagnosis is primarily clinical, though imaging, such as ultrasound (US) or, in uncertain cases, magnetic resonance imaging (MRI). Treatment is conservative, with spontaneous resolution generally occurring within four weeks [2]. 

In this report, we present the third and fourth cases of DSFC documented in Portuguese children, aiming to raise awareness of its diagnosis and management [2,4,5].

## Case presentation

Case 1

A 12-week-old boy presented to the pediatric emergency department with a swelling in the parieto-occipital region. The swelling, which was first noticed by the parents, had gradually enlarged over the past week.

His birth history was significant for a dystocic (vacuum-assisted) delivery at term. Fetal scalp electrodes were not used during labor, and no scalp swelling was observed in the immediate neonatal period. He had an Apgar score of 8 and 9 at one and five minutes, respectively.

The infant's medical history was otherwise unremarkable. He had no significant family history of hematological or connective tissue disorders, and there were no preceding injuries or trauma reported. He was exclusively breastfed and meeting developmental milestones appropriately for his age.

**Figure 1:**
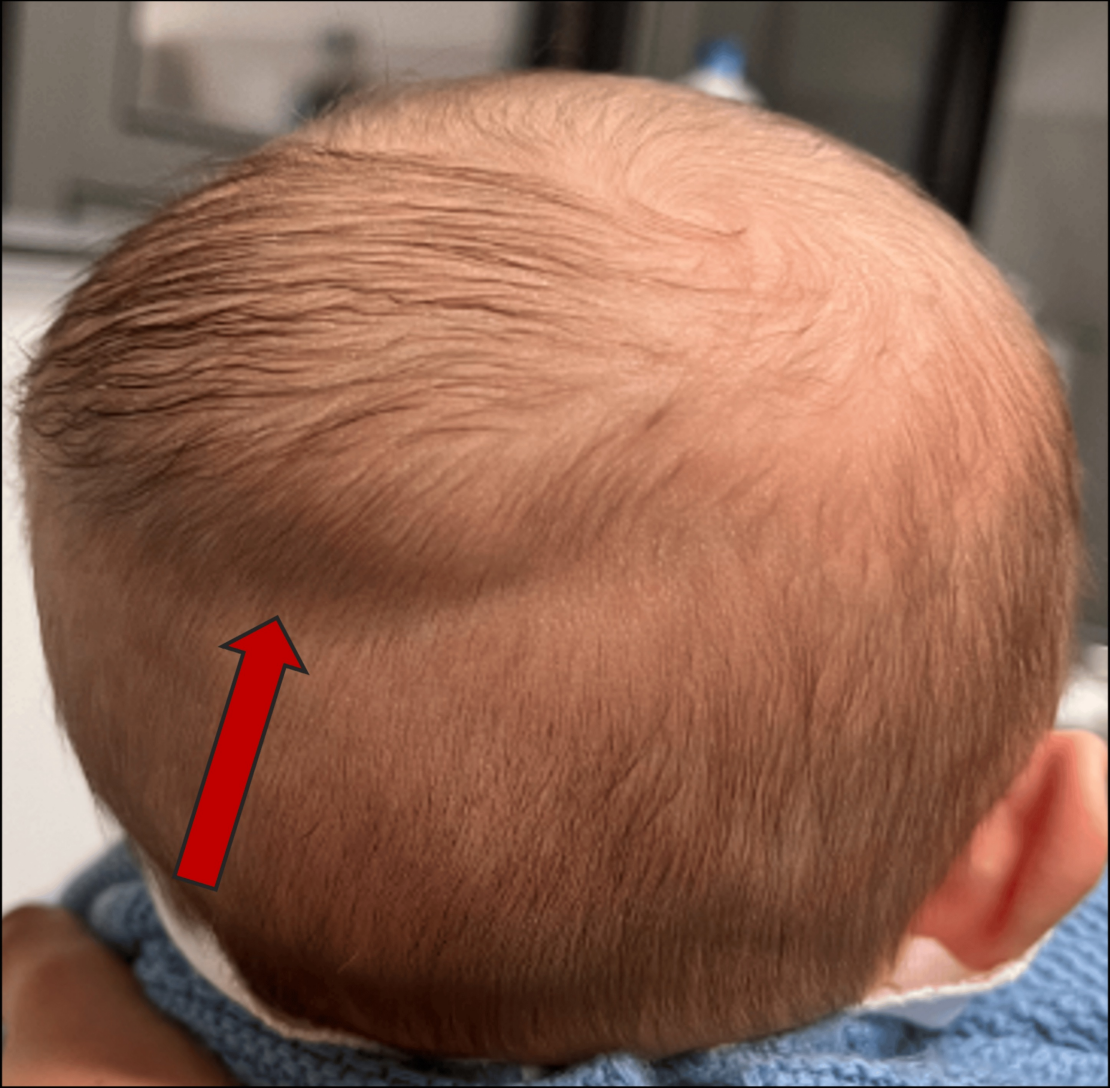
Fluid accumulation in the occipitoparietal region, with no signs of overlying scalp erythema or injury. The collection is not limited by the cranial suture lines. Red arrow: highlights the area of fluid accumulation.

On examination, the infant was alert, responsive, and hemodynamically stable with normal vital signs. The swelling was soft, non-tender, and fluctuant, crossing suture lines. There were no signs of overlying skin changes, such as erythema or bruising. The remainder of the physical examination, including cranial nerve assessment and a detailed neurological examination, was unremarkable. No other abnormalities were noted in other systems (Figure 1).

**Figure 2:**
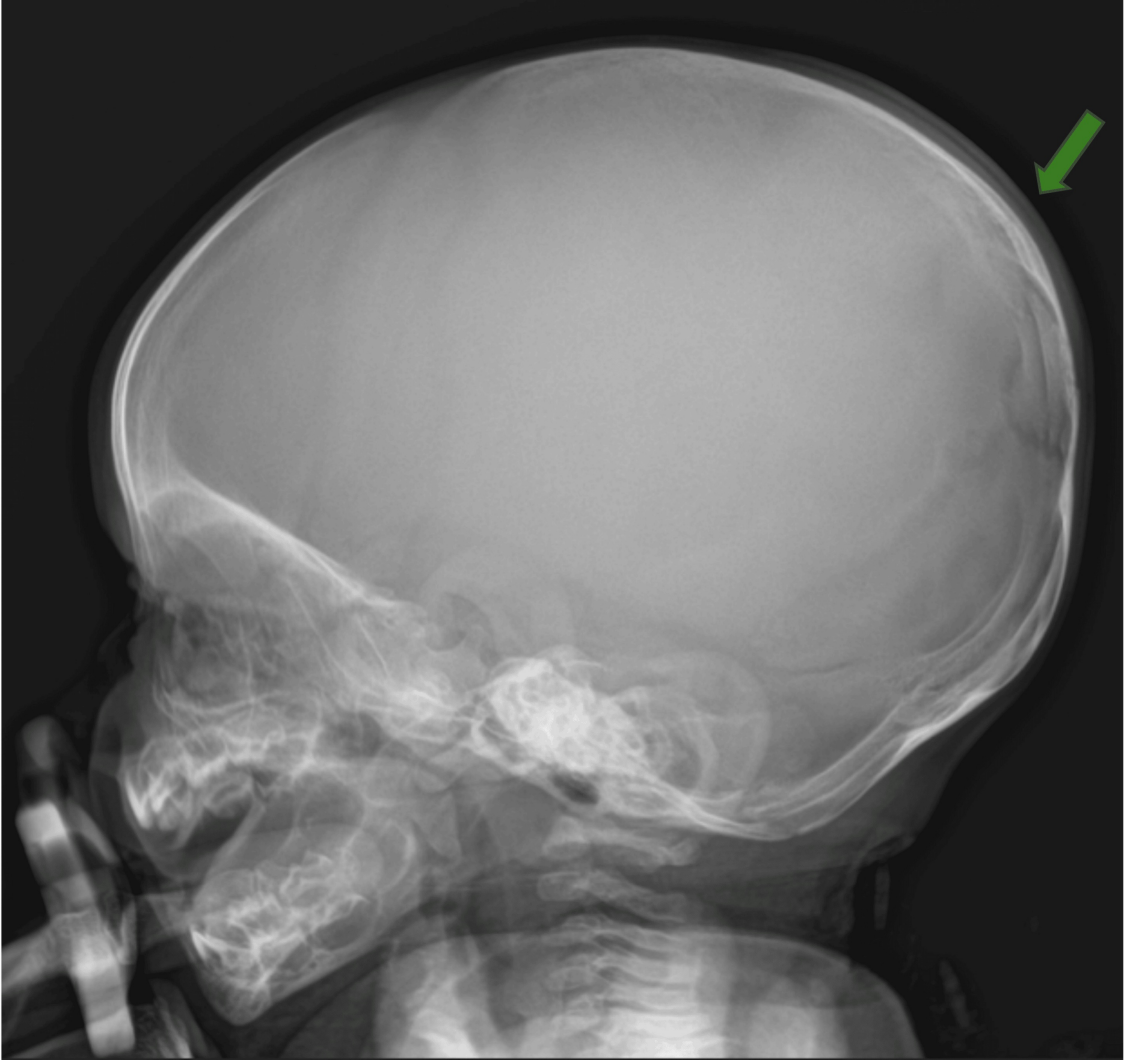
Cranial radiography performed in the emergency department shows no signs of bone fractures or masses. Green arrow: occipitoparietal region without evidence of bone injury.

**Figure 3:**
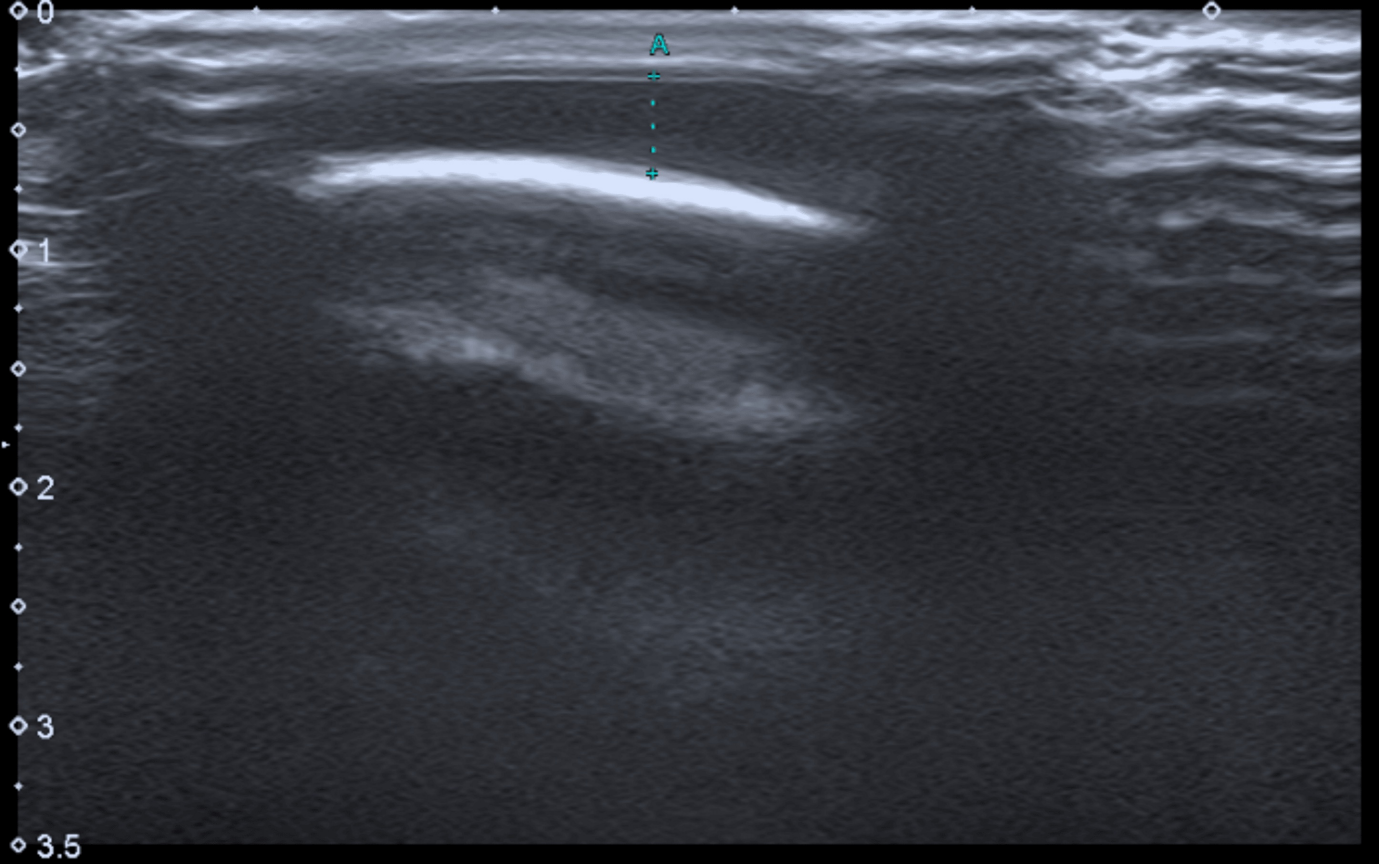
Cranial ultrasound - a small anechoic collection is identified (A), with simple ultrasound characteristics, not organized, in a subaponeurotic location.

Cranial radiography, performed initially, revealed no signs of skull fracture (Figure 2). Cranial ultrasound (US) showed an anechoic, avascular collection in the occipital subaponeurotic region, with no evidence of intracranial hemorrhage (Figure 3).

Case 2

A six-week-old girl, presented to the pediatric appointment with a three-day history of progressive swelling in the parieto-occipital region.

The infant was born at 41 weeks of gestation via a dystocic (vacuum-assisted) delivery. Her Apgar scores were 5 at one minute, 9 at five minutes, and 10 at 10 minutes, which required positive pressure ventilation. At birth, a small occipital cephalhematoma was observed, but it resolved spontaneously within five days.

She had no significant medical history, was healthy, and was meeting her developmental milestones appropriately. The family history was non-contributory, with no known hereditary conditions. There was no history of trauma or other significant incidents. The infant was being breastfed and was thriving well.

**Figure 4:**
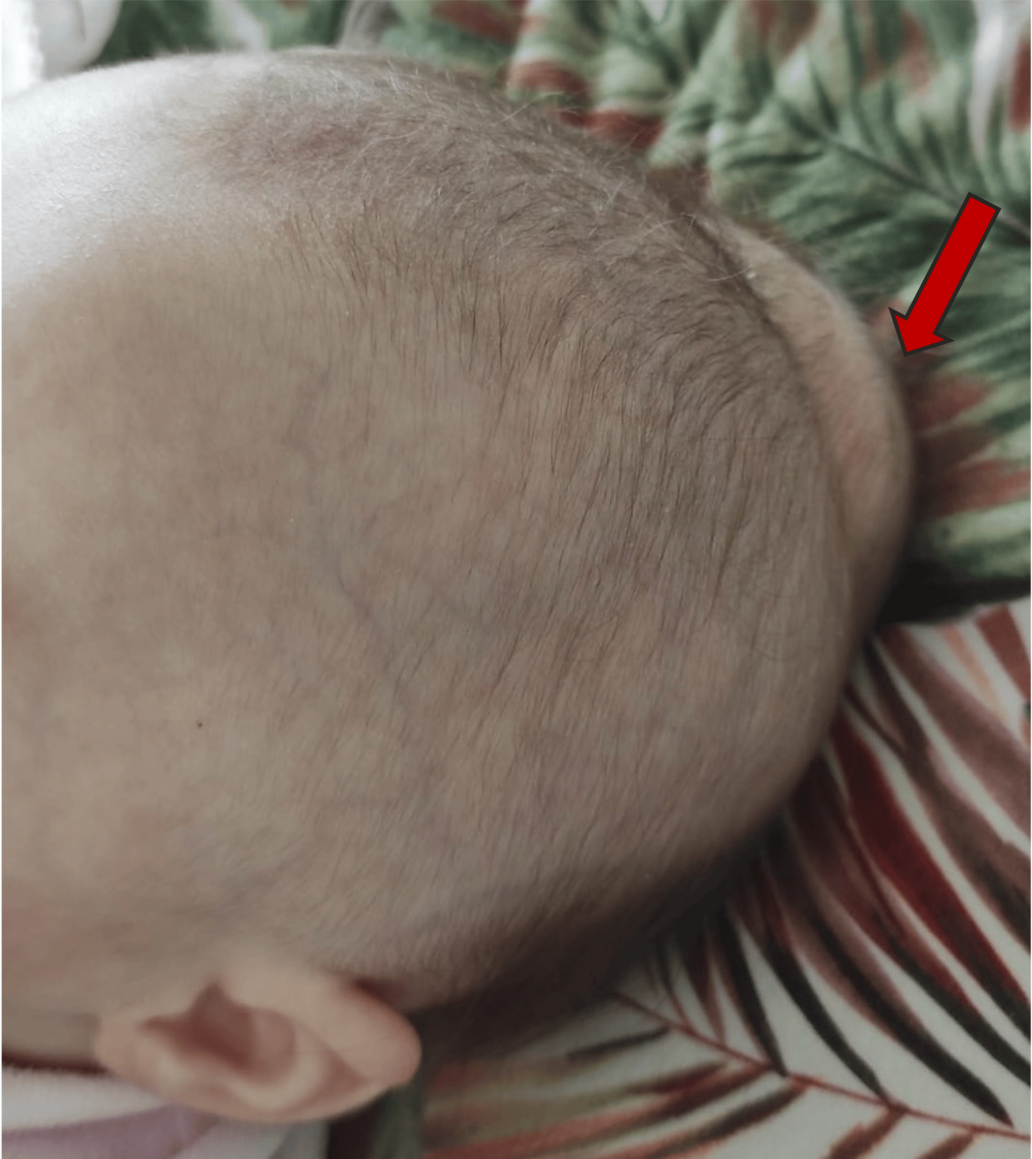
Fluid accumulation in the occipitoparietal region, with no signs of overlying scalp erythema or injury. Red arrow: highlights the area of fluid accumulation.

On examination, the infant appeared alert, active, and hemodynamically stable, with normal vital signs. The swelling was soft, non-tender, and fluctuant, measuring approximately 11.5 cm in length, and it was not limited by suture lines (Figure 4). There were no skin changes, such as discoloration or bruising, over the area of swelling. The remainder of the physical and neurological examinations were normal.

**Figure 5:**
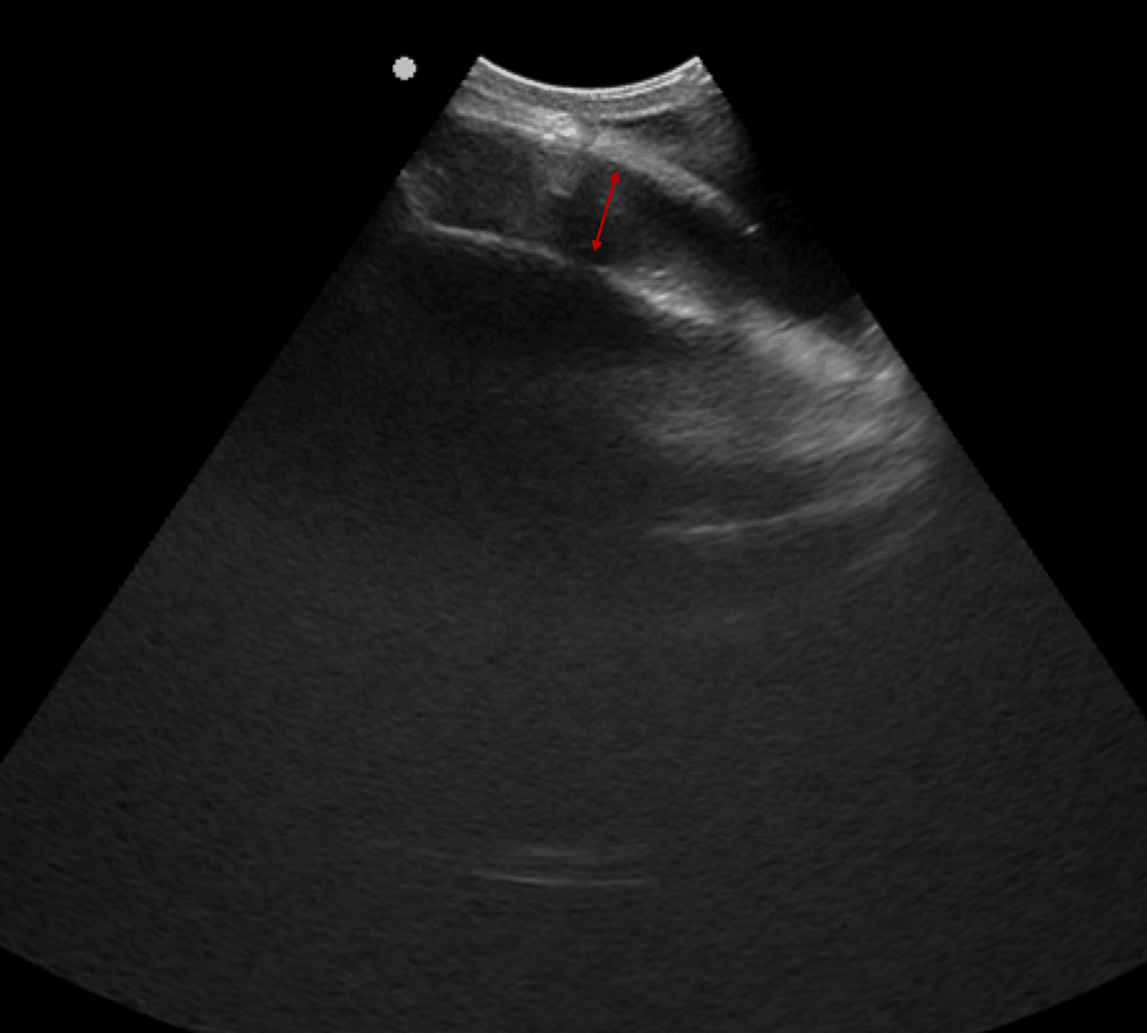
Cranial ultrasound -a small anechoic collection is identified, with simple ultrasound characteristics, not organized, in a subaponeurotic location. Red arrow: highlights the collection area.

Cranial ultrasound (US) showed an anechoic, avascular, symmetrical and highly compressible fluid collection, over the posterior fontanelle. No other relevant findings were noted (Figure 5).

In both cases, based on clinical and radiological findings, a diagnosis of DSFC was made. A conservative, expectant management approach was adopted, leading to a complete resolution of the swellings within one month, with no documented sequelae.

## Discussion

DSFC is an extremely rare condition with no clear etiology [1-4]. A 2024 systematic review by Cid Mendes et al. included only 67 cases [2]. It is characterized by the spontaneous onset of scalp swelling in neonates and infants. Consistent with other reports, our cases presented at six and 12 weeks of age, which is typical for DSFC. Both patients had no other medical issues or history of trauma. Physical examination revealed a large, boggy, and fluctuant fluid collection under the scalp. The collections were non-tender, and both infants appeared well.

Both patients were born via vacuum-assisted deliveries, a factor frequently associated with DSFC. Schoberer et al. detected β2-transferrin and β-trace protein, specific markers of cerebrospinal fluid (CSF), in the aspirated fluid from three infants diagnosed with DSFC [6-8]. This suggests that delayed CSF leakage into the subaponeurotic space may occur due to microfractures in the skull, potentially caused by a traumatic delivery or the use of fetal scalp electrodes [4,6]. Another proposed explanation involves the damage to the emissary veins linking the intracranial venous sinuses to the superficial scalp veins, or the obstruction of scalp lymphatic drainage within the subaponeurotic connective tissue layer during a difficult delivery [4,6-8].

While DSFC is primarily diagnosed based on clinical presentation, it is recommended to perform at least cranial soft tissue and transfontanellar ultrasound to exclude more serious conditions [2]. Ultrasound is safe, simple, cost-effective, and readily available. Typically, it reveals a mobile and hypoechoic fluid collection within the subgaleal space that extends across suture lines [2,3,6].

In both cases, complementary imaging studies were performed. In Case 1, cranial radiography was initially requested in the emergency department to rule out a fracture, even though there were no signs of head injury or history of trauma. Cranial ultrasound images were typical in both patients. If further delineation of the fluid nature is needed, magnetic resonance imaging (MRI) is considered the best modality but is generally unnecessary [3].

For both of our patients, conservative management approach was chosen, as unanimously recommended in the literature, with no interventions on the fluid collection. Other treatments, such as puncture aspiration, have not been shown to be particularly beneficial, and the potential risks, such as hemorrhage or infection, do not justify their use. The swelling resolved within four weeks in both patients, with no sequelae, consistent with most reports in the literature [4-6].

## Conclusions

DSFC is a rare but benign condition that presents with scalp swelling, typically weeks to months after birth. It is self-limiting, with spontaneous resolution usually occurring within four weeks. Awareness and early recognition of DSFC are crucial, as they can prevent unnecessary diagnostic procedures and interventions. These two cases contribute to the limited reports on DSFC and highlight the significance of considering this condition in the differential diagnosis of neonatal and infant scalp swelling.
